# The Functional Implications of Transanal Irrigation: Insights from Pathophysiology and Clinical Studies of Neurogenic Bowel Dysfunction

**DOI:** 10.3390/jcm13061527

**Published:** 2024-03-07

**Authors:** Gianna Rodriguez, Steven Kirshblum, Mai Thao, Mackenzie McArthur, Michael Camilleri

**Affiliations:** 1 Physical Medicine and Rehabilitation, Spinal Cord Injury Medicine, University of Michigan Health, Ann Arbor, MI 48108, USA; 2 Department of Physical Medicine and Rehabilitation, Rutgers New Jersey Medical School, Newark, NJ 07103, USA; skirshblum@selectmedical.com; 3 Coloplast A/S, Minneapolis, MN 55411, USA; 4 Division of Gastroenterology and Hepatology, Department of Internal Medicine, Atrium Health, Charlotte, NC 28204, USA; mackenziejarvis2424@gmail.com; 5 Division of Gastroenterology and Hepatology, Department of Internal Medicine, Mayo Clinic, Rochester, MN 55905, USA

**Keywords:** neurogenic bowel dysfunction, colonic motility disorder, transanal irrigation, neurogastroenterology, chronic constipation, fecal incontinence, impaired anal sphincters

## Abstract

Defecation function is negatively impacted in patients with neurogenic bowel dysfunction (NBD), who require effective bowel care for stool evacuation. NBD comprises fecal incontinence and/or constipation, which can reduce the quality of life and dignity. Transanal irrigation (TAI) is recommended by several clinical guidelines as the second-line treatment after conservative treatment and before surgical options are considered. As the only class in the second-line treatment with an established safety and efficacy profile, the mechanism of action of TAI has not fully been elucidated when administered through a rectal catheter with a balloon. This review examines the current understanding regarding the mechanism of action of TAI, with a focus on the pathophysiology of neurogenic bowel and irregular defecation. By understanding the functional implications of TAI, clinicians may be better able to integrate this modality into bowel care programs, especially for patients with NBD who have significant constipation due to delayed colonic motility and impaired stool emptying with loss of voluntary control of defecation, and those who are unresponsive to conservative treatment, including enemas.

## 1. Introduction

Neurogenic bowel dysfunction (NBD) is a complex condition following an injury or lesion to the spinal cord that affects the nerves involved in the control of gastrointestinal (GI) functions. The term NBD is commonly used to describe fecal incontinence (FI), constipation, or difficult stool evacuation experienced as a result of neurological disorders such as spinal cord injury (SCI), multiple sclerosis (MS), spina bifida, cerebral palsy, or Parkinson’s disease [1,2]. NBD can cause life-threatening complications, in addition to negatively impacting quality of life and an individual’s dignity [3,4]. 

NBD poses a significant burden for individuals who experience it. For example, individuals with SCI reported that bowel-related symptoms are one of the top five most important complications [5]. Moreover, 11% of re-hospitalizations of people with SCI result from GI complications [6]. In the MS population, the prevalence of NBD ranges from 39% to 73%, correlating to disability and duration of the disease, with individuals also ranking neurogenic bowel as one of their top concerns [7,8]. Similar concerns about neurogenic bowel are reported by adults with spina bifida [9].

NBD is complex, considering its multifactorial pathophysiology and varying symptoms. Recognizing the challenges of neurogenic bowel, in 2018, the Craig H Neilsen Foundation conducted a workshop to identify, target, and accelerate the research areas ready for clinical translation for SCI [10]. With the participation of several multi-disciplinary experts, bowel physiology was identified as a top priority to elucidate the effects and interventions on bowel function. Since this meeting, the Consortium of Spinal Cord Medicine Clinical Practice Guidelines on the Management of Neurogenic Bowel Dysfunction in Adults after SCI have been updated and several consensus statements have been developed inclusive of patients’ input [11,12,13,14,15]. More recently, Dietz et al. identified that meticulous attention to the patient is required during the first five years postinjury to achieve a successful bowel management plan that is sustained for 25 years [16]. 

Bowel programs for patients with NBD are individualized and comprehensive, with a goal to achieve effective bowel evacuation and prevent incontinence. Individualization is based on medical history, including GI symptoms, level and severity of neurological impairment based on the International Standard for Neurological Classification of Spinal Cord Injury and Autonomic Function following SCI, physical examination, images documenting fecal loading, and input from the patient and caregiver [12,13,14,15,17,18]. Comprehensive bowel care aims to achieve regular bowel movements with improved colonic transit and rectal evacuation, predictable defecation, completion of bowel care within 30–60 min, and increased independence. 

Several guidelines and consensus publications endorse a stepwise approach to bowel care, tailored to the individual [12,14,15,19,20]. Conservative treatment (also referred to as basic treatment or standard of care) includes diet and/or lifestyle modifications, oral laxatives, and rectal laxatives, possibly in combination with additional rectal evacuation assistance (i.e., digital anorectal stimulation). When the individual has insufficient or no response to conservative treatment, transanal irrigation (TAI) is recommended before surgical options such as appendicostomy with antegrade colonic irrigation, sacral nerve stimulation, and surgical ostomy are entertained. In the Consortium of Spinal Cord Medicine Clinical Practice Guidelines on the Management of Neurogenic Bowel Dysfunction in Adults after SCI, TAI is recommended in individuals with NBD who have insufficient results with conservative measures [15]. More recently, Magnuson et al. proposed an updated and simplified treatment algorithm for effective bowel care, reflecting clinical practice and commonly available treatment modalities, with TAI as a second-line step in treatment progression, especially for those who have insufficient response to conservative measures such as enema [14].

There is a gap in the literature regarding the mechanism of action (MOA) of the TAI device. Our aim is to review the current knowledge about the efficacy of TAI with an emphasis on the pathophysiology of neurogenic bowel dysmotility and abnormal defecation and to explore how a TAI device with a rectal catheter with a balloon acts as a prosthetic to promote successful stool evacuation for the treatment of NBD by functionally replacing impaired anal sphincter functions, stimulating peristalsis, and activating the rectoanal inhibitory reflex. Understanding how TAI works will facilitate the customization of bowel programs and new interventions for NBD.

## 2. Neuroanatomy and Physiology of Functional Defecation

Defecation is a fundamental physiological process resulting in fecal evacuation. Its voluntary control allows for continence. Both defecation and continence are heavily dependent on the coordination and integration of multiple systems such as the neural and muscular components of the GI tract.

### 2.1. Somatic and Autonomic Nervous Systems and Enteric Nervous System

The somatic and autonomic nervous system (sympathetic and parasympathetic) and the enteric nervous system (ENS) work in concert to govern secretion and motility in the colon and anal sphincter function. The ENS is the intrinsic nervous system comprising several plexi, the major ones being the submucosal (Meissner) plexus and the myenteric (Auerbach) plexus. The submucosal plexus is sparse in the stomach and prominent in the small and large intestines, where there are large or small ganglia interlinked by internodal strands containing hundreds of axons. The myenteric plexus extends throughout the digestive system, from the pharyngoesophageal junction to the internal anal sphincter (IAS). These two plexi have different functions: the submucosal plexus mainly controls mucosal secretion and blood flow, and the myenteric plexus located between the circular and longitudinal layers of the muscularis propria is primarily involved in the coordination of motility patterns. The interstitial cells of Cajal (ICC) and platelet-derived growth factor receptor α (PDGFRα)-positive fibroblast-like cells, sometimes collectively referred to as the gut “pacemaker” cells, are present within the plexi as networks throughout the GI tract. These pacemaker cells are coordinated with the extrinsic autonomic nervous system to determine the specific frequency, velocity, and direction of propagation of colonic motor patterns [21,22,23]. The ENS also regulates the peristaltic reflex, which responds to intrinsic afferent signals from the intraluminal content and is responsible for the efficient propulsion of bowel contents via neural circuits and motor function [24]. With the extrinsic input of sympathetic and parasympathetic nerves, the modulated enteric reflexes generate proximal to distal propulsion of colonic contents and relaxation of the IAS [25]. In general, parasympathetic input is excitatory (promotes contraction) to non-sphincteric muscle and inhibitory (promotes relaxation) to the sphincters, whereas sympathetic input is inhibitory (promotes relaxation) to non-sphincteric muscle and excitatory (promotes contraction) to the sphincters [26]. The extrinsic parasympathetic nerves to the distal gut that are vulnerable in neurologic disorders emanate from Onuf’s nucleus (a distinct group of neurons in the ventral part [lamina IX] of the anterior horn of the sacral region of the human spinal cord), course along the sacral S 2–4 roots and reach the colon, rectum, and external anal sphincter (EAS). It is important to note that parasympathetic nerves give rise to ascending intracolonic nerve fibers that extend for a variable distance through the wall of the mammalian colon to stimulate colonic peristalsis [27]. 

Adding to this complex intrinsic and extrinsic innervation, the gut microbiota have putative effects on colonic motility, at least in part through the synthesis of short-chain fatty acids and the biotransformation of bile acids, including the deconjugation of glycine and taurine and the dehydroxylation of primary to secondary bile acids. The microbiota play a role in maintaining normal defecation and continence, and having a balanced and diverse gut microbiome is considered healthy. The influence of the gut microbiota on colonic function occurs throughout life [28].

### 2.2. Four Phases of Defecation

Four distinct temporal phases of defecation occur to maintain continence or facilitate defecation. During the **preparation (basal) phase**, the colon prepares its content for eventual expulsion via several homeostatic functions (e.g., mixing the content; water and electrolyte fluxes; formation of short-chain fatty acids; solid stool; etc.). Cyclic motor patterns, most likely generated by ICCs, along with other motor patterns facilitate colonic motility, including high-amplitude propagated contractions (HAPCs), which are responsible for mass movements in the colon and occur about six times per day, typically following meals and on awakening in the morning [29]. The propagation of the cyclical motor complex and HAPCs in the colon appears to be similar to esophageal peristalsis and involves the suppression of inhibitory (neuronal nitric oxide synthase) motor neurons and the release of mucosal 5-HT, resulting in 5-HT activation of myenteric neurons, leading to muscular excitation [30]. The cyclic motor complex pattern is hypothesized to act as an “intrinsic brake” to prevent the untimely flow of colonic contents and promote continence [31,32]. Continence is also achieved through the resting tone of the anal sphincter and pelvic floor musculature by the voluntary contraction of the somatically innervated EAS. During the **pre-expulsive phase**, a series of antegrade propagating contractions move the intraluminal content towards the rectum for preparation for evacuation, allowing for rectal filling and distension to elicit the rectoanal inhibitory reflex (RAIR), which relaxes the IAS and generates a conscious defecatory urge through rectal mechanoreceptors and sensory receptors in extrarectal tissues and the pelvic floor. Using a magnetic tracking system, Nandhra et al. determined that the distal transit of a capsule from the descending colon to the sigmoid colon takes 30–60 min [33]; scintigraphic studies also show that the rectum and sigmoid function act as a volitional reservoir retaining stool until it is feasible to evacuate [34]. It is during this phase that defecation can be voluntarily deferred via contraction of the EAS and retrograde motility to return rectal contents to the sigmoid colon. Defecation proceeds to the **expulsive phase** if appropriate; the antegrade propagating contractions increase in both frequency and amplitude and this is associated with the urge to defecate. The relaxation of the IAS and pelvic floor musculature as well as voluntary EAS relaxation occur after initiating the RAIR. Reflex relaxation of the IAS can also be initiated with intentional rectal distention using an inflated balloon [35,36]. While the cyclic motor pattern is inhibited, the intraluminal content enters the rectum and anal canal for evacuation. In conditions associated with rectal evacuation disorders in the absence of NBD, there is prolonged retention of stool in the rectosigmoid region [37]. During the **end phase**, defecation is terminated, and the basal rectoanal pressure gradient is re-established by the contraction of the anal sphincter and pelvic floor to achieve continence. 

## 3. Pathophysiology of Neurogenic Bowel

Disruption of descending autonomic pathways, especially parasympathetic nerve activities, leads to the abnormalities in the ENS observed in NBD. Neurologic dysfunction leads to FI and/or constipation and is based on the anatomical level and completeness of the disorder of the spinal cord or roots in relation to conus medullaris. Accordingly, neurogenic bowel is divided into two patterns based on the anatomical relation of the lesion to the conus medullaris, which is the location of the terminal portion of the spinal cord and the anterior horn cells of the sacral segments, S2–S5. The study of the spectrum of spinal cord injuries has facilitated our understanding of neurogenic bowel. Thus, findings relating to NBD in SCI can be applied to NBD with other etiologies, such as Parkinson’s disease and MS. In this review, we will use the terms infraconal SCI to infer injury or lesions involving upper and lower motor neurons and peripheral sacral nerves that are likely to be damaged. We will use the term supraconal SCI to refer to injury or lesions above the conus medullaris. 

### 3.1. Hyper-Reflexic Bowel and Impact on Defecation

Hyper-reflexic neurogenic bowel patterns are consequences of supraconal spinal cord lesions, just as upper motor neuron injury in the somatic nervous system leads to exaggerated reflexes. Loss of inhibitory input from supraconal centers in the central nervous system leads to an increased resting rectal tone. Though not reaching statistical significance in a study by Krogh et al., the amplitude of RAIR in SCI individuals with supraconal lesions was numerically greater compared to that in normal individuals [38]. In the same study, rectal tone was significantly higher than normal in patients with acute and chronic supraconal lesions but significantly lower in patients with acute and chronic conal/cauda equina lesions, and the proportion of subjects with single giant rectal contractions was significantly higher than normal after acute supraconal spinal cord injury but not after acute conal/cauda equina lesions. Whether colonic function is also compromised depends on the level of injury. Thus, spinal lesions above L1 are associated with abnormal motor responses in the left colon, whereas injuries above T5 affect the right and left colon [39]. In addition, both the resting anal sphincter tone and RAIR contribute to an overactive segmental peristalsis and underactive propulsive peristalsis [40]. The combination of hyper-reflexic patterns and physiological responses leads to constipation as the main clinical symptom. This is exacerbated by the hyper-reflexic EAS and lack of defecation urge. A study using radiopaque markers found that total colonic transit time in individuals with chronic supraconal lesions was longer than that of normal individuals (mean, 3.93 days versus 1.76, respectively; *p* < 0.05; based on the Mann–Whitney test); the slowest colonic transit occurred in the transverse and descending segments [41]. Prolonged colonic transit has been confirmed in other neurological diseases, such as spina bifida, Parkinson’s disease, and MS [42,43,44]. In the context of defecation, the basal phase is impacted by prolonged colonic transit. The effects of impaired anal sensation on the pre-expulsive phase result in FI, manifested by involuntary stool leakage or overflow incontinence secondary to fecal impaction [45].

### 3.2. Hyporeflexic Bowel and Impact on Defecation

Hyporeflexic neurogenic bowel patterns occur when infraconal lesions are present. Damage to the spinal cord conus, sacral nerves, axons of cauda equina, and/or pudendal nerves result in diminished rectal tone and reduced amplitude of RAIR in addition to EAS weakness leading to FI. The interrupted bidirectional relationship between extrinsic neural control and enteric systems is manifested by absent anocutanous, bulbocavernosus, and other lumbosacral reflexes along with missing external sphincter and sacral dermatome innervation via the somatic nervous system and parasympathetic nervous system. In fact, both the internal and external sphincters are hypotonic and function poorly due to the lack of innervation. Adding to the already compromised structures, the phases of defecation are also affected. Krogh et al. observed that SCI individuals with chronic conal lesions had a prolonged colonic transit time compared to control individuals (mean of 3.61 days versus 1.76, respectively; *p* < 0.05; based on the Mann–Whitney test) in a study using radiopaque markers [41]. A prolonged colonic transit time during the basal phase may lead to increased mucosal fluid absorption, producing firmer and less frequent stool [45]. A hypotonic anal sphincter coupled with a lack of anal sensation negatively impacts the pre-expulsive phase of defecation, eventually resulting in FI. The inability to seal the rectum during the end phase of defecation because of an impaired anal sphincter and pelvic floor musculature further exacerbates incontinence [46,47]. 

## 4. The Role of TAI in Managing NBD

TAI is a second-line intervention for patients with NBD and is often recommended after patients experience insufficient results with standard or conservative care [12,14,15,20]. This review article focuses on the system with a rectal catheter with a balloon. The choice of device, TAI delivered through a rectal catheter with a balloon or cone, should be individualized to the patient’s condition, dexterity, and expectations [20,48]. In this section, we review the major characteristics of TAI administered with a rectal catheter with a balloon and identify the clinical outcomes and benefits of TAI beyond reducing episodes of FI and constipation for individuals with neurogenic etiology of bowel dysfunction. We shall document those characteristics while focusing on the rectal catheter’s mechanism of action (MOA). Thorough and comprehensive systematic review articles covering the safety and efficacy of TAI have been published [49,50].

TAI devices with rectal catheters with a balloon include a pump, water bag, and control unit. The catheter is inserted into the rectum and the balloon is inflated to seal the rectum. Lukewarm tap water (36–38 °C) is then introduced into the colon using the pump and control unit. When the balloon is deflated and the catheter removed, the result is bowel emptying. Examples of FDA-approved TAI devices with a rectal catheter with balloon are Peristeen™/Peristeen™ Plus by Coloplast (Humlebaek, Denmark) and Navina Classic Irrigations systems™ by Wellspect (Molndal, Sweden). 

The foundation for TAI with a rectal catheter with a balloon for the treatment of NBD in adult patients was laid by Christensen et al. [51]. In their randomized controlled trial, SCI patients with NBD were randomized to conservative treatment (i.e., best supportive bowel care without irrigation) or the TAI procedure. After 10 weeks, there were significant improvements in bowel symptoms with TAI compared to conservative bowel management (Table 1) when assessed with different scales including the NBD Score, which is a validated questionnaire based on symptom score for clinical assessment of colorectal and anal dysfunction in SCI individuals [52].

Christensen et al. also determined the TAI group spent less time on their bowel management and used the device at least every two days. The TAI group also had fewer urinary tract infections (UTIs); no further investigation was conducted in the study to determine a definite explanation for the fewer UTIs. Since then, several studies from different centers and investigators report TAI is similarly effective in adult individuals with SCI, spina bifida, multiple sclerosis, and NBD with other etiologies [53,54,55,56,57,58]. 

Patients gained other clinical benefits from using TAI in addition to their improvement in FI and/or constipation and improved quality of life. In a multicenter prospective study, 36 patients who were unsatisfied with their conservative bowel care used TAI with a rectal catheter with a balloon, and 28.6% of the patients reduced or eliminated the need for pharmaceuticals, with improvement in FI and/or constipation [59]. They also had fewer episodes of leakage of feces and spent less time on stool evacuation. In another study, patients spent less than 30 min on total bowel management when using TAI, which may be attributed to the reduced duration and frequency of defecation [57,60]. These studies suggest TAI users gain time along with the predictability of evacuation.

A systematic review on the effect of TAI on bowel function included 27 eligible studies including 1435 individuals (3 randomized controlled trials, 1 non-randomized trial, and 23 observational studies with 70% of the studies of excellent or good methodological quality, albeit including limited data) [49]. Results showed an improvement in bowel function among patients with NBD and other bowel etiologies, with some studies showing improvement in quality of life. However, discontinuation rates were high, ranging from 8 to 57%. Reasons for discontinuation were inefficacy, pain during application, practice problems, and lack of satisfaction. Side effects included abdominal cramps, anorectal pain, chills/shivering, nausea, dizziness, and sweating. While these were common, they were equally prevalent among comparative treatments.

A recent review evaluated the literature on TAI as a complement to standard bowel care for NBD and included 19 studies that showed reduced difficulties associated with defecation, episodes of incontinence, time needed for evacuation and bowel care, increased general satisfaction with bowel habits and quality of life, and decreased level of dependency [61]. There were practical problems (e.g., leakage of irrigation fluid, balloon explosion) and side effects (e.g., abdominal pain/discomfort, anorectal irritation, anal/rectal bleeding, bowel perforation). Discontinuation was relatively common mainly due to practical problems, adverse events, and unsatisfactory effects. Users, including caregivers, reported practical problems, and compliance was not always easy to achieve. 

Bowel perforation is a rare complication of TAI. Christensen et al. reported 49 reports of bowel perforation related to TAI in 2015, corresponding to an average rate of bowel perforation of two per million procedures [62]. The majority (67%) of the events occurred within eight weeks of starting treatment. With long-term use, the risk is less than two per million procedures. The authors noted that careful patient selection, patient evaluation, and proper training of patients are critical to the safety of TAI application. 

TAI may be performed by the individual or by a caretaker, and successful outcomes are influenced by a range of factors. Optimal patient selection is one of the key factors, and this is related to male gender, mixed constipation and fecal incontinence symptoms, and prolonged colonic transit time [63]. It is also dependent on the patient’s motivation and psyche. Emmanuel et al. recommended a “trial-and-error strategy for the introduction of TAI should be applied” coupled with direct supervised training to establish individualized parameters for irrigation [20]. Systematic approaches to troubleshoot practical problems and minimize side effects are instrumental to successful outcomes [20,48]. 

Compliance with TAI is another key factor to sustaining successful TAI outcomes. When assessing the use of TAI at home, 62.5% patients were still using TAI after a mean of 2.6 years, and they were highly satisfied with TAI despite technical problems [60]. A predictive factor for compliance with TAI was the progress of training [64]. The implementation of a systematic education to the patient and caregiver within the first three months is crucial to sustaining the treatment long-term. 

### Proposed MOA of TAI in Triggering Effective Evacuation

**Figure 1:**
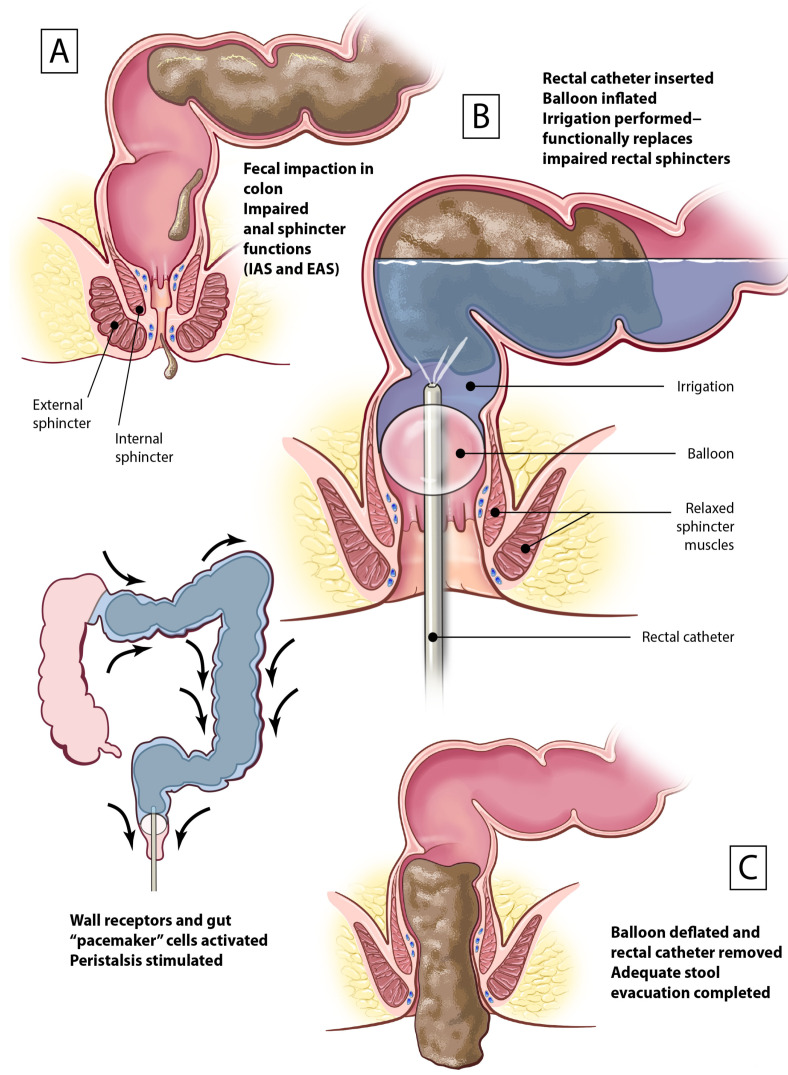
Proposed mechanism of action of TAI. (**A**) Gut with NBD which has fecal impaction and loss of anal sphincter functions (i.e., internal anal sphincter [IAS] and external anal sphincter [EAS]). (**B**) Transanal irrigation delivered via a rectal catheter with a balloon. The balloon is inflated, replacing the function of the impaired anal sphincters, and peristalsis is stimulated in the colon. The balloon also triggers the rectoanal inhibitory reflex, causing IAS relaxation. (**C**) The balloon is deflated, and evacuation is completed following removal of the rectal catheter.

A remaining knowledge gap regarding TAI is the comprehensive understanding of the mechanism to support defecation and continence. In NBD, anal sphincters malfunction and colonic motility is impaired, causing inadequate defecation. Existing information suggests a TAI system composed of a rectal catheter with a balloon may exhibit a prosthetic function, particularly when the balloon inflates in the rectum, replacing the function of impaired anal sphincters. Balloon inflation is necessary to retain intracolonic content and deflation supports the stool evacuation function. The device facilitates pressurized water that is introduced into the colon via the rectum; the water is retained by sealing the outflow with the inflated balloon, leading to the activation of intact stretch receptors and intrinsic sensory nerves in the colonic wall and the stimulation of the intact intrinsic neuromuscular apparatus to stimulate peristalsis, propelling the colon contents distally [58,65]. In parallel, the rectal catheter stretches the rectum and activates RAIR (whether its amplitude is high or low), relaxing the IAS to facilitate evacuation when the balloon is deflated and the mechanical obstruction is removed [36]. Figure 1 shows the proposed MOA of the rectal catheter with a balloon in the context of transanal irrigation. By functionally replacing the function of the sphincters and stimulating peristalsis along with triggering the relaxation of IAS, TAI has a mechanistic impact on the phases of defection, more specifically re-establishing the basal and expulsive phases. 

The proposed MOA may help explain why the TAI procedure is effective for both hyper-reflexic and hyporeflexic bowels. In hyper-reflexic bowel, the rectal catheter may augment the hypertonic/dyssynergic anal sphincter. When pressurized water is introduced via the inflated rectal balloon catheter, it stretches the luminal wall and activates gut pacemaker cells. The activation of intrinsic neuromuscular mechanisms interferes with the hyper-reflexic bowel, which is characterized by overactive segmental peristalsis and underactive propulsive peristalsis. Meanwhile, in hyporeflexic bowel, the rectal balloon catheter functionally replaces the hypotonic/flaccid anal sphincters and promote peristalsis. 

Furthermore, the MOA may help explain the reduced constipation and FI episodes after using TAI. An earlier study by Christensen et al. used scintigraphy to demonstrate that water introduced via TAI was able to reach the right colic (hepatic) flexure, which was associated with fecal clearance from the rectosigmoid and most of the descending colon, where feces are stored [66]. Based on the proposed MOA, peristalsis stimulation could be a factor in removing feces from these areas. The rectum is also emptied. Considering that the rectum is filled with feces by the colon as a precursor to evacuation, without a filled rectum, FI cannot occur (although flatus incontinence or leakage of mucus could still occur) [67]. In summary, evacuation is promoted with TAI so that patients spend less time on bowel management and have fewer episodes of constipation and FI.

A clinical study by Ethans et al. supports the proposed MOA. Ethans et al. determined an improved colonic transit time using a Metcalf (radiopaque marker transit) method after administering TAI with a rectal balloon catheter [65]. In their study, colonic transit time improved by 22% (from mean 84.1 h at baseline to mean 65.4 h at follow-up) after using TAI for 10 weeks in individuals with cauda equina syndrome (CES). From a clinical perspective, the improvement in colonic transit time with TAI came closer to that of individuals without NBD, that is, 35.0 ± 2.1 h (mean ± standard error, with estimated range of 20–52 h) compared to the transit time of CES individuals of 86.64 h [68]. The patients also had improvements in bowel symptoms and quality of life. The authors stated that water introduced by the rectal catheter with a balloon emulsifies the inspissated stool and stimulates colonic peristalsis resulting in evacuation. 

In another study, Ascanelli et al. studied the effects of TAI on FI and constipation in MS individuals [69]. Quality of life and NBD scores both improved after using TAI for these patients. Furthermore, MS with intestinal disorders simulating NBD showed a correlation of enrichment of pathobionts with increased intestinal permeability and pro-inflammatory cytokines, and depleted microbial taxa were associated with health. With TAI, gut microbiota diversity increased while the proportion of pathobionts decreased. Taken together, these data indicate an added benefit of remodeling the gut microbiota beyond improving bowel dysfunction. 

## 5. Conclusions

In this review, we synthesized the functional implications of TAI when delivered by a rectal catheter with a balloon with existing information. As a second-line therapy, understanding its mechanism of action can bolster the utilization of TAI in bowel management programs for patients with NBD, especially for those who experience inadequate results with enema or other conservative treatment options. Such inquiries into TAI’s mechanism of action shed light on its unique characteristics and may help identify future interventions.

TAI is a safe and effective treatment for NBD, reducing fecal incontinence and/or constipation while improving quality of life. There are side effects, including bowel perforation, which is a rare complication. Discontinuation of TAI may occur; however, implementing a systematic education of TAI application to patients and/or caregivers, especially within the first three months, is key to long-term adherence to TAI. Furthermore, patient selection and patient evaluation are critical to the clinical success of TAI.

There is still much to learn about the pathophysiology of NBD in different neurologic diseases. Development of better treatment and future interventions for NBD relies on our understanding of this. Furthermore, more research is required to investigate how NBD specifically alters the GI microbiome, neurotransmitters, various receptors, immune and inflammatory responses, humoral and secretory factors, etc. Together, these efforts will ensure that with increasingly individualized bowel management programs, there are effective treatment options available. 

## Figures and Tables

**Table 1:** Overview of the bowel symptoms and quality of life from Christensen et al. [51].

Scale	TAI Group Scores	Conservative Treatment Group Scores	*p*-Value
Cleveland Clinic Constipation *	10.3	13.2	0.0016
St. Mark’s Fecal Incontinence *	5.0	7.3	0.015
NBD Score *	10.4	13.3	0.048
Quality of life	6.3	4.2	0.00009

* Low scores indicated less severe symptoms; comparisons of means at termination were performed using 2-sided Student *t* test, or for non-parametric data, Wilcoxon 2-sample test and Χ2 test for absolute frequencies.

## Data Availability

No new data were created or analyzed in this study. Data sharing is not applicable to this article.
